# Social, Economic and Human Capital: Risk or Protective Factors in Sexual Violence?

**DOI:** 10.3390/ijerph19020777

**Published:** 2022-01-11

**Authors:** Paola Ilabaca Baeza, José Manuel Gaete Fiscella, Fuad Hatibovic Díaz, Helena Roman Alonso

**Affiliations:** 1 School of Psychology, School of Juridical and Social Sciences, Miraflores Campus, Universidad Viña del Mar, Valparaíso 2520000, Chile; 2 Career of Sociology, Faculty of Social Sciences, Campus FACSO UV, Universidad de Valparaíso, Valparaíso 2340000, Chile; jose.gaete@uv.cl; 3 Career of Psychology, Faculty of Social Sciences, Campus FACSO UV, Universidad de Valparaíso, Valparaíso 2340000, Chile; fuad.hatibovic@uv.cl; 4 School of Social Work, Faculty of Social and Economic Sciences, Campus San Miguel, Universidad Católica del Maule, Talca 3466706, Chile; hroman@ucm.cl

**Keywords:** sexual violence, social capital, human capital, economic capital

## Abstract

In Chile, studies on protective factors and risk factors for sexual violence are limited and very few have incorporated analysis of different types of capital (social, economic, human) as social resources in the protection against sexual violence. The objective of this research is to evaluate to what extent the stock of different capitals act together, as either protective or risk factors in sexual violence in different interpersonal environments. The sample consisted of 1665 women between 15 and 30 years of age (M = 23.47, SD = 4.41). Artificial neural network analysis and social network analysis were used. The nodes representative of human and economic capital have a protective role of low relevance due to their position in the network, while the nodes of social capital acquire a structural relevance due to the central positions of the network. It is concluded that the structural social capital of neighborhood networks constitutes the main protective factor for sexual violence in all areas, and in turn, the structural social capital of networks with non-significant others was the main risk factor in sexual victimization.

## 1. Introduction

The definition of sexual aggression according to UN WOMEN includes the following aspects: (a) It is an act of physical or sexual violence against a person, (b) It occurred without the person’s consent, (c) The victims and perpetrators must not be gender-specific, and (d) It is a violation of a person’s bodily integrity and sexual autonomy [1]. In Chile, according to the Sexual Crimes Unit of the National Prosecutor’s Office, 51 people a day were victims of sexual violence during 2010 [2]. Eight years later, there was a significant increase in this type of crime by 24.81%, being the highest registry of complaints since the beginning of the Criminal Procedure Reform in Chile, and of whom the main victims were women [3]. In this context, it is relevant to delve into this type of violence due to the serious biopsychosocial consequences of having lived this experience, such as reproductive health problems or sexual and psychological dysfunctions [4,5]. Even more so, considering that the victims of sexual violence do not report the experience, especially when the perpetrator is the partner.

According to the latest report from the World Health Organization (WHO), the prevalence of sexual violence in intimate relationships ranges from 5% to 47% [6]; prevalence rates that have alarmed the various nations and organizations working on this type of aggression. In this regard, international policy on violence against women has recognized this serious social problem and has been working to prevent violence for at least 75 years [7]. International studies on sexual aggression worldwide found that 7.2% of women worldwide reported having been sexually assaulted by a non-partner in 2010 [8]. The international labor organization reports that at least 4.8 million women are victims of some type of sexual exploitation [9].

In this line, the #metoo movement of 2017 highlighted the sexual assaults experienced daily by hundreds of women in the workplace, the impact of which brought about the creation of a series of regulations on labor matters to promote a harassment-free work environment in different organizations around the world [10,11]. In addition to this figure, we must consider “the dark figure” of sexual aggressions, which according to several international studies is estimated to be between 10 to 20.5 times higher than that reported [12,13,14]

Other investigations carried out in Chile in the context of university students report prevalences ranging from 24.4% to 51.9% [15,16,17]. Despite the valuable information provided by these studies, the current state of knowledge is still limited because, in the first place, data provided by the National Prosecutor’s Office is only based on complaints made by the victims or a third party and do not take into consideration the hidden numbers of those who do not report this crime.

Second, most research on sexual violence has focused on the relational context of a partner and/or friends, and mostly on samples of university students, excluding people who study in other non-university higher education institutions (e.g., technical-professional) and those who work or are engaged in domestic duties. Third, most of the investigations have not asked about these experiences in other areas, such as those occurring in the public sphere and/or work environment, which represent an important gap, making it difficult to compare figures at national and international levels. Additionally, timely intervention and the design of specific prevention programs for this type of violence has been lacking.

Under this outlook, a considerable number of investigations have focused mainly on evaluating risk factors associated with sexual violence, (e.g., attitudes supporting rape, alcohol and/or drug use, childhood abuse, negative influence of peers and witnessing domestic violence, among others) thus supporting relevant information to address the problem [18,19,20]. However, very few studies have focused on evaluating protective factors of sexual violence. The knowledge base in this area is extremely limited, even though evidence exists about the role that protective factors play in reducing the negative impact of sexual violence [21]. For example, social support has been found to be a protective factor for the perpetration of sexual violence even when the risks of prior sexual victimization and alcohol use were controlled, although only in same-sex couples [22]. Another study found that men with greater knowledge of sexual consent and adequate family functioning were less likely to engage in sexual assault in the university context [23]. Likewise, there is evidence that emotional health and connection with the community were protective factors for the perpetration of male sexual assault [24]. In Chile, studies on protective and risk factors are limited, and those that do exist demonstrate that attending religious activities and living with parents are related to a lower probability of being a victim of sexual violence [19]. However, this study has a reduced sample in the Chilean context.

Although these studies are useful in understanding those factors that prevent the perpetration and victimization of sexual violence, most of them focus on protective factors associated at individual levels (e.g., knowledge of sexual consent, emotional health), and do not consider environmental protective factors that can go towards helping victims of sexual violence. In addition, of those investigations that have evaluated social environmental protective factors—such as community connection—only adolescents or university students have been the focus of study, and the main results of those studies show the importance of attending religious activities in preventing sexual victimization [24,25,26,27,28]. As youth who participate in these activities tend to engage in less risky behaviors (e.g., alcohol consumption), their peers are likely to engage in similar behaviors [24,25,26,27,28].

Other research outside Chile reports the role of empathy as a moderator in the relationship between certain risk factors such as approval of forced sex between peers and sexual aggression [29]. Likewise, gender inequalities in a society have been related to a greater probability of sexual victimization even for men [30].

In this context, some research shows that beyond individual factors (e.g., attitudes supporting rape, use of alcohol and/or drugs, mental health, among others) in the victimization of sexual violence, characteristics such as not belonging to a social group, socioeconomic disadvantages of the neighborhood and difficulties in accessing different social networks, among others, would be associated with a greater risk of experiencing violence, including sexual violence [31,32].

Along these lines, very few studies have incorporated an analysis of different types of capital as social resources in the protection against sexual violence [33]. This aspect is relevant, since experiencing sexual violence can lead to social isolation due to distrust and shame of the situation, reducing social functioning of the victims [33]. Under this panorama, evaluating to what extent the different types of capital (social, economic and human) could be a protective factor for these experiences becomes relevant.

The first of these is social capital, a multidimensional concept that has been investigated in its association with violence in society. The definition of social capital refers to resources available to a person through social networks and belonging to groups through social norms of trust and reciprocity that facilitate help and cooperation for mutual benefit [34,35]. Thus, social capital can be categorized into structural social capital, referring to stable networks of interpersonal relationships and belonging to a social group; or cognitive social capital, which includes norms, values, attitudes and beliefs that promote collective behavior of cooperation and mutual benefit driven by trust among its members [36,37]. In this sense, the beliefs associated with sexual stereotypes create a basis for expected behaviors for the sexes. Both types of social capital are interrelated, that is, beliefs (cognitive social capital) create expectations about people’s behavior, creating a support of trust in the social network in which they participate (structural social capital).

According to the theory of social capital, communities with higher levels of social capital would have a lower prevalence of violence of all kinds. Although there are few studies that have evaluated the role of social capital in sexual violence, some report that for each increase in one point in the index of social capital of women there was a 30% decrease in the probability of experiencing intimate partner violence [38]. Likewise, community cohesion and informal social control were protective factors for intimate partner violence in communities where this type of violence was unacceptable [31]. Similarly, support services for women victims of violence often help them develop relationships of trust and establish networks that enhance their social capital [39].

This aspect is relevant since it has been found that cities with low social capital also have a lower development in gender equality, which contributes to the maintenance of traditional gender stereotypes that reproduce inequalities based on patriarchal conceptions [36]. It has also been found that cities with high social capital and less acceptance of traditional gender roles have a lower prevalence of violence against women [40]. In this regard, it has been shown that beliefs associated with gender inequalities and favorable attitudes towards violence against women are attenuated by high social capital. In this sense, gender inequality is a risk factor for violence, because it exposes women to less cohesive and weaker networks and, therefore, less social capital, especially when these networks are gender homophilic [41].

In addition to social capital, there are other types of capital that have been associated with experiences of sexual violence, including economic capital, that of property rights, salary and all sources of income, being the best convertible capital and base for obtaining income, and human capital, understood as the accumulated level of education, training, information and productive skills of the population [42,43]. In this regard, the authors have recognized that having high economic and human capital is associated with a lower probability of being exposed to experiences of sexual violence [44,45,46].

However, although the initial conception of social, economic and human capital has been attributed to a series of benefits and positive consequences, it should be pointed out that they do not necessarily constitute a protective factor since they could also have negative consequences. For example, social capital can be negative when it has the ability to restrict people’s freedom (e.g., belonging to criminal gangs where there is a high level of trust, a high level of support and close relationships) [47] and can discourage personal initiative [48]. Regarding cases of sexual violence, social capital would not be a protective factor in those communities where this type of violence is justified or ignored. For example, in societies with high rates of gender inequality and whose social norms validate certain risk factors (e.g., rape myths) associated with sexual victimization [30]. In this case, the influence of social (cognitive) capital on intimate partner sexual violence is probably greater than economic and human capital.

Furthermore, in the same way that social capital can be a risk factor, it has also been found that human and economic capital could—although to a lesser extent—facilitate the prevalence of different types of violence [49,50].

In this context, the current study aims to evaluate to what extent, given Chilean realities and peculiarities, the stock of the different capitals act, jointly, as protective factors or, on the contrary, as risk factors when evaluating the experience of sexual violence in the public, work, educational and intimate partner relationships of Chilean women.

The specific objectives are: 

To evaluate to what extent social, economic and human capital act as protective and/or risk factors in sexual victimization in the public sphere in Chilean women.

To evaluate the extent to which social, economic and human capital act as protective and/or risk factors in sexual victimization in the workplace in Chilean women.

To evaluate the extent to which social, economic and human capital act as protective and/or risk factors in sexual victimization in the educational setting in Chilean women.

To evaluate to what extent social, economic and human capital act as protective and/or risk factors in sexual victimization in intimate partner relationships in Chilean women.

The hypotheses of the present research are:

**Hypothesis** **1** **(H1).** *It is expected to find that women with low social, economic and human capital report more experiences of sexual victimization in the public sphere*.

**Hypothesis** **2** **(H2).** *It is expected to find that women with low social, economic and human capital report more experiences of sexual victimization in the workplace*.

**Hypothesis** **3** **(H3).** *It is expected to find that women with low social, economic and human capital mention more experiences of sexual victimization in the educational setting*.

**Hypothesis** **4** **(H4).** *It is expected to find that women with low social, economic and human capital mention more experiences of sexual victimization in intimate partner relationships*.

## 2. Materials and Methods

### 2.1. Participants

Responses were analyzed from the public database of the structured survey entitled “Violence against women in the field of domestic violence and in other spaces” (public database: ENVIF-VCM) carried out by the Undersecretary of Crime Prevention in the Ministry of the Interior and Public Security between 2019 and 2020. The target population included women between the ages of 15 and 95, residing in urban areas of all 16 regions of Chile.

The sample design was stratified randomly by multi-stage clusters. For the primary sampling unit, population by city blocks was considered; for the secondary sampling unit, we considered households and finally, for the tertiary sampling unit, we studied the people living in the houses. The sample consisted of 6775 women between the ages of 15 and 65. The sampling error was 1.2%.

For the purposes of this research, a subsample of young women was selected, whose ages were between 15 and 30 years, totaling a final sample of 1665 cases. The choice of this age range corresponds to the fact that young people in Chile, also defined by different international and national organizations [51,52], are between the ages of 15–30. The surveyors were properly trained and the surveys were conducted using a tablet. Table 1 shows the sociodemographic characteristics of the participants. It should be clarified that in the civil status, the concept of civil union agreement (AUC) is a solemn contract entered into between two persons of the same or different sex who share a household, with the purpose of regulating the legal effects derived from their affective life in common, of a stable and permanent nature.

### 2.2. Instruments

The questionnaire used was created by the Undersecretary of Crime Prevention, which includes questions addressing the sociodemographic characteristics of the participants and their families, norms, values and attitudes towards male and female roles, prevalence of violence (psychological, physical and sexual) and participation in community networks. According to the enquiries presented in the ENVIF-VCM-OFFICIAL questionnaire, the following variables were constructed for the study.

#### 2.2.1. Sexual Violence

Sexual assault was evaluated in the different settings where the experience occurred: public, work, or educational environments and within a couple relationship, sometime in their lifetime during the last twelve months. The answer alternatives for all these questions was yes or no.

Sexual violence in the public sphere was understood as violence experienced in the street, public transport, in a square, places of entertainment or parties, sports venues, etc., by an acquaintance or a stranger, and were included in questions P70, P70.3, P70.4 and P70.4. An example of this type of question: “Was she forced to have sexual relations or was there intent to force her to have sexual relations?”

Sexual violence in the workplace was considered to be any experience of violence in the workplace by a boss or coworker, and were included in questions P80, P80.c, P80.e, P80.f. An example of this type of question: “Was she forced to have sex or was there intent to force her to have sex?”

Sexual violence in the educational environment was considered to be any experience during student life and attempted by a director, teacher or classmate. Included were questions P90, P90.4, P90.6, P90.7, P90.8. An example of this type of question: “Tried to have sex or tried to force her to have sex?”

Sexual violence in the field of intimate partner relationships included questions P132.A, P132.a, P132.b, P132.c, P132.d, P132.h. An example of a question of this dimension was: “Were you physically forced to have sex when you didn’t want to?” 

#### 2.2.2. Social, Economic and Human Capital

Social capital: To evaluate structural social capital, questions associated with participation in networks (Q65, Q66 and multiple-choice) were considered. Question Q65 was “How often do you see or communicate with...?” The response alternatives were: immediate family, people I consider family, friends, neighbors, religious communities, others. Question Q66 was: “If you have a problem, do you ask someone for help?” The response alternatives were dichotomous yes and no. In the case of an affirmative answer, they were then asked to whom they turned for help, repeating the same alternatives of answers from question Q65. 

For cognitive social capital, questions associated with norms, values and attitudes towards male and female roles (questions P64 to P64.13) were considered. Two examples of questions in this section would be: (i) The woman should take care of the children instead of the man; (ii) A woman should have sexual relations with her husband/partner, even if she does not want to. The response alternatives were on a Likert-type scale where 1 was strongly disagree and 5 was strongly agree. Cronbach’s alpha was 0.74.

Economic capital: Questions of socioeconomic status and economic dependence were considered. The socioeconomic level variable was constructed in the public database and the response alternatives were: high, medium and low. The economic dependence variable was also created in the public database and the response alternatives were: low economic dependence, medium economic dependence and high economic dependence.

Human capital: Questions associated with the educational level of the woman and of her partner were included. The question for both cases was: What is the highest educational level attained by you? What is the highest educational level attained by your current partner? The answer alternatives ranged from no education to postgraduate studies.

### 2.3. Data Analysis

To define relationship networks between the variables, the application of “artificial neural networks” was used with the JASP software, both for the dependent variables (sexual violence in different settings) and for variables representing the different types of capital (social, economic and humane). Initially, the technique does not seek to specify the existence of a latent variable that clusters or is common to the set of variables, rather, to identify partial or complete correlations between the variables [53], demonstrating the significance of each dyadic correlation with respect to the rest of possible correlations that can be developed by the rest of the nodes of the network. Therefore, a significant relationship between the nodes (variables) is justified when the covariance between both nodes is not exceeded by the covariance with the other nodes in the network [54,55].

Thus, the choice of this technique allows us to evaluate the joint action of independent and dependent variables at the same time. Additionally, it allows us to overcome the restrictions of parametric multivariate analysis (normal distribution, homoscedasticity, etc.) and, also, the possibility of including variables with different measurement levels in the analysis [53]. 

In particular, and for the purposes of this work, the “IsingSampler” estimator was used, developed mainly by Epskamp [56] from Van Borkulo et al. [57], which allows for the analysis of non-numerical variables. Specifically, the relationships between the variables are defined through logistic regressions for each of the variables that are evaluated, and where each variable is, on some occasions, the dependent variable of the rest of the variables. Thus, the existence and strength of a relationship (positive or negative) between the nodes (j and k) is verified through the parameters (β jk) of said regressions [58,59,60].

The second technique used was the analysis of social networks using the UCINET 6 software. Thus, a transformation is applied to the resulting neural network to achieve two “mode 2 networks”. The first mode 2 network, defined from relationships with positive coefficients, was called the “risk network”, since the increase in one variable implies the increase in a type of violence. On the contrary, the mode 2 network formed exclusively by negative relationships was defined as a “protection network”, since an increase in one variable implies a decrease in a type of violence. Subsequently, algorithms were applied to obtain the centrality of the nodes in the network and, with it, the structural relevance (considering the structure of the network itself) of both the factors and the areas of sexual violence [61].

## 3. Results

### 3.1. Descriptive Analysis Prevalence of Sexual Aggression

The results of the prevalence of sexual aggression found were as follows: the experience of sexual aggression suffered in the public sphere was 17%, in the workplace 5.9%, in the educational sphere 6.4% and 12% within the couple’s relationship.

Table 2 and Table 3 present the descriptive statistics for structural and cognitive social capital.

### 3.2. Definition of the Neural Network

**Figure 1:**
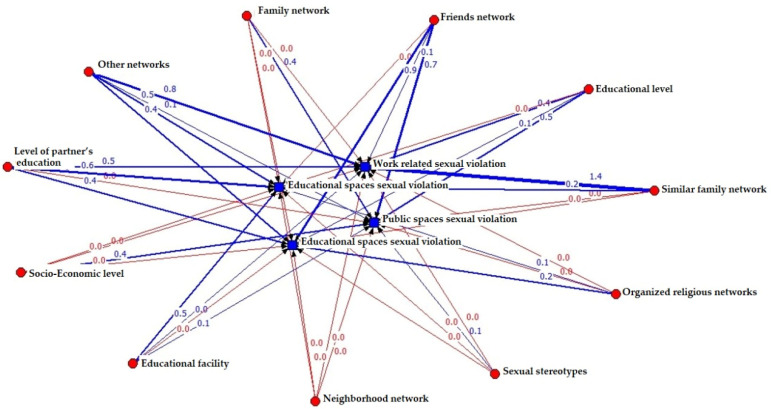
Neural network (result) between factors (social, economic and human capital) and areas of sexual violence. (Note: for graphic purposes, negative values are represented with a value of 0.0 and with a red line).

The formation of the neural network shows the relationship between the capitals (social, economic and human) and the different areas of sexual violence (result network), as shown in Figure 1. As can be seen, the disposition of all the variables and their link to one another is exemplified by differently colored ties: the red ties are negative relationships and the blue ties are positive. The former indicate a negative average regression coefficient between the nodes (variables), that is, both variables are explained inversely in their interaction. On the contrary, positive relationships—both variables—are explained in the same direction in their interaction. The mass for both types of relationships indicates the relevance of the average coefficients in such a way that the greater the mass, the greater the direct or inverse relationship, as the case may be. 

Similarly, the position of the nodes in the network is defined by the Fruchterman–Reingold algorithm [62] which, in general terms, calculates the force of attraction (given by the frequency of positive relationships) between neighboring nodes and the repulsion force (given by the frequency of the negative relations) between all the nodes of the network. The position of the network nodes is defined according to the sum of opposite forces [62].

Finally, the quantification of positive or negative relationships between factors and areas of sexual violence is presented in Table 4. From this table, we define protection networks and risk networks (structure of negative relationships in red and positive relationship in blue). They are defined as this type of relationship because negative relationships imply that a higher factor level leads to a lower level of violence with which it is related or determined, that is, a negative causal relationship. On the contrary, a network of only positive relationships implies that the increase of a variable or factor indicates an increase in the prevalence of the type of sexual violence with which it is related.

As can be seen, there are factors (types of capital) that act on areas of sexual violence in different ways. On the one hand, there are factors that, when increasing, generate a decrease in one or more areas of sexual violence. For example, as the socioeconomic level factor increases, it generates a decrease in the prevalence of sexual violence at work, within the couple and in the educational field. On the contrary, the higher the socioeconomic level, the higher the prevalence of sexual violence in the public sphere.

**Figure 2:**
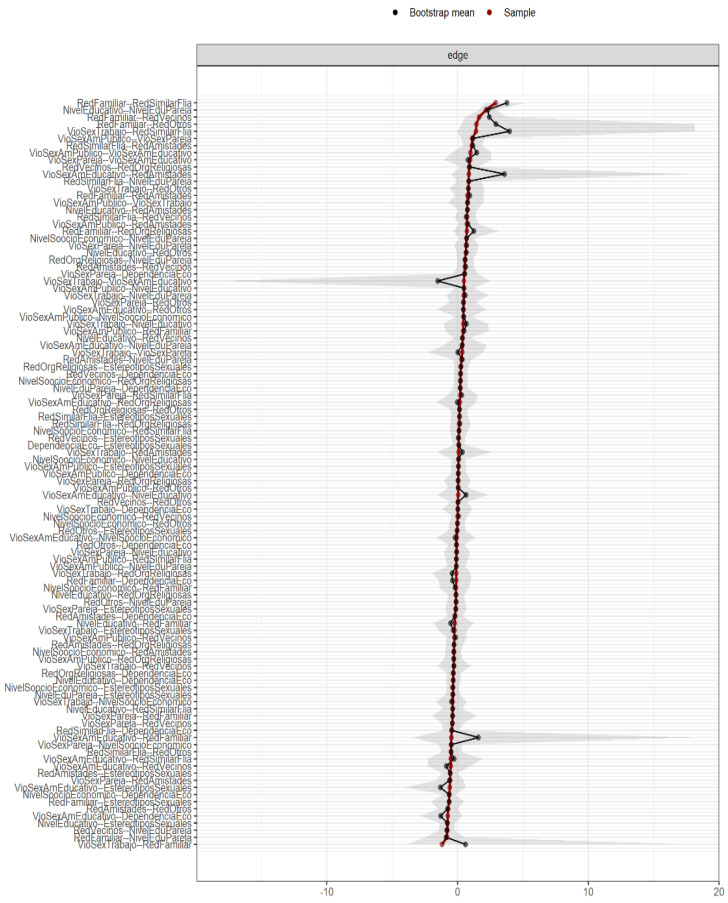
Neural network goodness-of-fit indicator.

However, it is necessary to evaluate the good fit of the results delivered by the estimated neural network. To this end, we applied the “non-parametric bootstrapping” procedure as recommended by Epskamp [53], implementing it to 1000 simulated samples in order to evaluate whether said samples generate regression coefficients similar to those obtained in the resulting network, as shown in Figure 2. The superposition of the values shows (red and black lines) a concordance between the coefficients of the simulated networks (bootstrap mean = black line) and the mean coefficients of the result network (sample = red line). Therefore, it can be shown that the results obtained by the neural network are, for the most part, stable and have a good fit [53].

### 3.3. Lattice Analysis

#### 3.3.1. Protective Networks

**Figure 3:**
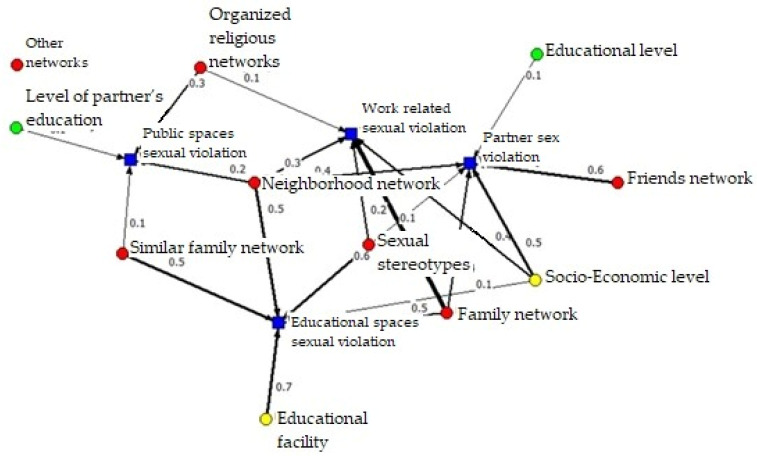
Protection network between capital and sexual violence.

As indicated above, to analyze the protection network, the negative relationships between the factors (capitals) and the different areas of sexual violence were selected. The resulting network is shown in Figure 3. This is a network that is defined as a “Mode 2 Network” [61], since it exemplifies the relationship between two types of nodes: factors (capitals) and areas of sexual violence. As can be seen in this network called protection, the nodes are positioned on the plane, given the relational relevance they have in the network. Thus, for example, factors such as networks of neighbors, family members and sexual stereotypes have a greater “centrality”, both due to the number of relationships they have with the nodes “sexual violence” and because of the strength of said relationships.

Consequently, and depending on the types of capital, we can observe that the representative nodes of human capital (green nodes) have a protective role, but of low relevance, given that they are positioned on the periphery of the network. Something similar happens with the representative factors of economic capital (yellow nodes). On the contrary, to a large extent, the representative nodes of social capital acquire a structural relevance given that they occupy central positions in the network and that, except for the network of friends and other networks, they have prevalence protection in more than one area of sexual violence. A quantification of this can be evidenced in the indicator “Degree” in Table 5, which quantifies the number of direct relationships between the nodes of the network [61].

As a representative of cognitive social capital, sexual stereotypes also reach a high centrality. In this case, however, their relationship is the opposite, given that the higher the level of beliefs in sexual stereotypes, the lower the prevalence of sexual violence in the workplace, educational space and within a couple. In the same way, if we consider the indirect relationships between nodes through the indicator “Closeness” in Table 5, we see that among the six factors with the greatest closeness to all the nodes of the network are, again, factors related to social capital. Therefore, and with the exception of the socioeconomic level, we can say that of the three capitals, social capital seems to have a greater protective function. 

With regard to the areas of sexual violence, we can observe that sexual violence within the couple and in the educational sphere are the types of violence most “lessened” by these three types of capital, while sexual violence in the public sphere obtains a lower protective action of all types of factors, both direct (Degree) and indirect (Closeness).

#### 3.3.2. Risk Networks

**Figure 4:**
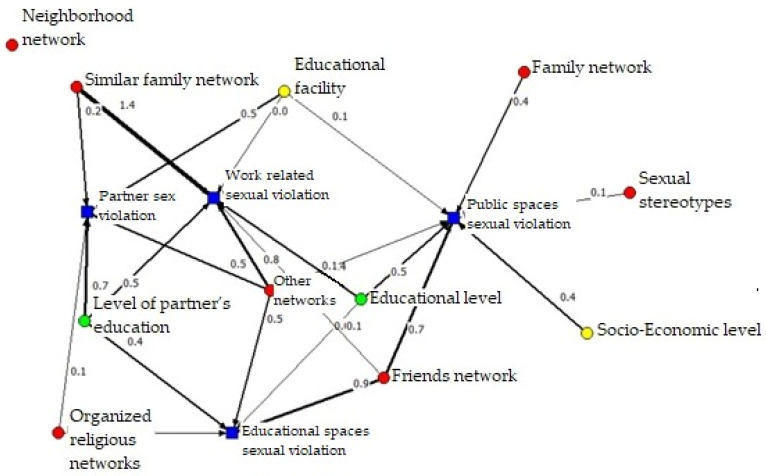
Risk network between capital and sexual violence.

The risk network is built from positive relationships where factors enhance the prevalence of sexual violence in different settings, evidenced in Figure 4. It is observed that the network defined in mode 2 between factors and areas of sexual violence shows how the variables of human capital reach a greater centrality compared to the protection network and, between the two, enhance the four types of violence. An extreme situation is observed in the “Other Networks” factor, which in itself promotes sexual violence in all areas; an antagonistic case to the network of neighbors that, as previously observed, does not promote any area of sexual violence. These variables, together with the friend network factor, are the ones that are most relevant (Table 6), both directly (Degree) and indirectly (Closeness). Finally, it should be noted that along this same line of analysis, factors of social capital occupy the last places in the centrality indicators, which confirms their potential for protection against sexual violence in different areas. Sexual violence in different areas is, as expected, exposed to these factors with differentiated enhancers. Thus, undercapitalization tends to promote sexual violence more in the public sphere, that is, sexual violence at work and in public spaces (Table 6).

## 4. Discussion

The main objective of the study was to evaluate to what extent the stock of different capitals (social, economic and human) act together either as protective or risk factors in sexual violence in different interpersonal areas (relationship, employment, educational and public).

### 4.1. Social, Economic and Human Capital as a Protective Effect for Sexual Violence

We found that participation in networks with neighbors would have a protective effect on sexual violence in all settings. Our findings are consistent with other studies showing that structural social capital, such as social ties with neighbors, contributes to reducing intimate partner violence, including sexual violence [63,64]. The explanation for these sexual violence findings could be related to social pressure and punishment the aggressors would receive in case they practiced said violence. For example, in Chilean prisons, sexual offenders also suffer attacks of this kind by other inmates as a form of revenge or social justice for the act committed. This situation is known to Chilean society, especially when the victims of sexual offenders are minors [65,66]. Similarly, communications and neighborhood meetings within community networks are likely to serve as a vigilance against sexual violence [67]. As pointed out by O’Campo et al. [68], we found that in a community, social cohesion (communication and networks between neighbors) and informal social control had important effects on the cessation of violence within the couple.

In line with the aforementioned, the routine activity theory (PAR) also helps us to explain this phenomenon. The PAR points out that one of the conditions for a crime to occur—whatever its nature—is the absence of a guardian capable of deterring it. A capable guardian is understood as any person or thing that discourages the possibility of committing a crime. In this sense, neighboring networks could constitute a guardian capable of discouraging the possibility of committing sexual violence [69].

Having a higher socioeconomic level and participation in family networks demonstrated a protective effect for sexual violence at work, in education and within the couple. Results that align with other investigations show how economic capital, specifically at a high socioeconomic level, is related to lower levels of violence [64,70]. Economic inequality not only implies a greater probability of experiencing violence, but also decreases social trust both at an individual and general level [71,72]. As is well known, Chile is one of the countries with the greatest socioeconomic inequalities in the world, having an impact on access to various social services and an exposure to different risk factors for violence. In this sense, it is not surprising that women with a higher educational level have a greater protection network, such as the family. Thus, frequent contact with family members has been associated with lower prevalence rates of violence [73]. Various studies show that maintaining good communication and emotional bonding within the family, in addition to participating in shared activities with this group, prevents exposure to different health risk experiences such as violence [74,75].

In addition, we must remember that perpetrators often use social isolation as a strategy to exert greater control over their victims in order to prevent them from seeking help to leave an abusive relationship [76,77], and in this sense, frequent contact with the family could be seen as a threat to the aggressor in situations of violence.

Another protective factor for sexual victimization in the three aforementioned areas (work, education and partnerships) is associated with cognitive social capital, specifically with the acceptance of norms, values and attitudes related to traditional gender roles and stereotypes. This result is contradictory with the findings of various investigations demonstrating that acceptance of stereotyped gender attitudes is associated with a high prevalence of sexual violence [74,75,78,79,80,81]. The explanation for these results could be due to the fact that many times women who suffer this type of violence tend to justify the violent behavior of their partners, rationalizing these experiences. Furthermore, when a woman agrees with statements such as: “A woman should have sexual relations with her partner, even if she does not want to”, it is very likely there is no discussion with her partner regarding consent to sexual activity, making her desire about whether or not to participate in this activity invisible. However, we know that engaging in non-consensual sexual activities through coercion or by responding to the traditional female role undermines women’s self-esteem. In addition, Chile is still a conservative country in the process of transition from traditional to egalitarian roles, and this aspect could influence awareness regarding women’s sexual rights.

Participation in networks of religious communities and frequent communication with people who consider themselves family members—while not being family members—are protective factors for sexual violence in the educational and public sphere (family similar networks) and public and work sphere (networks of religious organizations). Similar results by other researchers showed a significant decrease in experiences of violence among those who regularly attend church [28,81,82]. However, these results were framed in the context of couple relationships. Despite this, we consider that the role of religious communities in the prevention of sexual violence would be based on spiritual support for women who have lived these experiences. Although in recent decades the participation of people in religious institutions and activities has decreased in Chile, the last census found that 67% of Chileans consider themselves Catholic, with women being the main transmitters of these beliefs. In this sense, participation in religious communities in a Catholic society such as Chile is of vital importance in family and individual life. Additionally, religious leaders emphasize that the Catholic Church contributes to the protection of traditional values in order to prevent family disintegration, which they designate as a cause of violence in society [25]. Finally, we must not forget that sexual violence according to religious communities is an offense to God that is condemned by the Church, unlike other types of violence (physical, psychological, etc.) whose causes are understood by different social factors in the community [25]. On the other hand, other researchers have shown that increased religiosity is associated with lower rates of sexual victimization or perpetration [28,83].

The importance of participating in networks with people who consider themselves family members alludes to the trust that one has in these types of relationships. Therefore, it seems reasonable to assume that those people who have this type of network and trust each other will organize to condemn different crimes such as sexual violence in public and/or educational spaces. This aspect has been evidenced by other authors who emphasize that high social participation in certain communities could serve as a formal control and achieve low levels of victimization in society [82]. In addition, the ideas that people have about sexual violence who make up this network could have a protective effect. In this sense, the concept regarding sexual violence in these areas (public and educational) is widely condemned by society, and therefore, it is not surprising that participation in networks with people who consider themselves to be family members is one of the main sources of protection [83].

Finally, some factors of structural social, economic and human capital have an individual protective effect for sexual violence in certain settings. Specifically, we found that high educational level of the partner had a protective effect for sexual violence only in the public sphere. This result could be associated with one of the characteristics of Chilean society when framed in its great social inequalities. In Chile, having a high educational level as human capital is associated with access to various goods and services, including frequenting and connecting with homogeneous communities (high human capital), which implies, for example, living in neighborhoods with lower crime rates. In addition, a clear wage gap exists between men and women in Chile, in favor of men, having the same educational level. Having a partner with high human capital implies accessing a better quality of life and therefore, a lower risk of experiencing sexual assault in the public sphere.

The high educational level of women along with their participation in networks of friends are protective factors only for sexual violence within a couple, as has been found in previous studies whose main results show that connections in friend networks reduced the probability of sexual violence by up to 46% [84,85]. These results suggest that friendships could serve as guides in risk situations, which would increase awareness by women and rejection of a sexual violence event. In this sense, Pérez-Trujillo et al. [85], propose that connection with friends reduces the risk of sexual victimization because friends could dissuade a possible aggressor due to the social intervention they could generate in the event of that experience.

Economic dependence also has a protective effect for sexual violence only in the educational sphere. This element can be explained not only by the existence of financial support, but also in that pursuing higher education is a protective factor for different experiences threatening mental health, such as sexual violence. In addition, access to higher education has a significant economic cost for families (between EUR 2680 and 7503 per year) in Chile, where the average monthly salary is around EUR 482. Therefore, most people in Chile need financial support to pursue higher education, which generally comes from a family member.

### 4.2. Social, Human and Economic Capital as Risk Factors in Sexual Victimization

Participation in networks with others outside the inner circle of women explains sexual victimization in all spheres (public, work, educational and couples). This suggests that these links (with others) do not necessarily develop trust, cooperation and mutual help, essential elements of social capital, because many relationships—with others—could be motivated by utility, i.e., have a utilitarian function. In addition, consistent with the above, Chile has high levels of interpersonal, systemic and institutional distrust that could explain these results [86].

Economic dependence is positively related to sexual violence in the public sphere, at work and in the couple. Results consistent with previous research show that economic dependence on another exposes women to different experiences of violence, due to the lack of freedom to leave an abusive relationship or a precarious job [87].

The higher educational level of the partner was also positively related to sexual violence at work, in education and in the couple. Results consistent with another study carried out in a Latin American context documented that sexual offenders in relationships tended to have a higher educational level [50]. It is likely that higher educational level is related to greater power in the relationship, a characteristic that has been associated with sexual aggression, and the same is true for polyvictimization, that is, a woman is likely to experience multiple forms of victimization in different contexts [88,89,90].

Friends are risk factors for violence in the public sphere, at work and in education. Results consistent with other studies show that friend networks have been associated with greater violence [33]. These results could be understood by considering that the frequency of sexual violence in these areas is lower than in couple relationships, therefore, the silence, guilt and shame experienced by the victim in these cases could be an element that would explain these results. Another factor that could influence these results could be related to the nature of these ties and the relationship with peers who have risk factors for sexual victimization, such as alcohol abuse. In addition, we must not forget the role cognitive social capital plays on the acceptance of traditional attitudes and beliefs of gender roles, attributing blame to the victim in situations of sexual violence in these areas (public, work and educational) and transmitting the message that they get the punishment they deserve for their likely provocative behavior.

Higher educational level of women was positively associated with sexual violence in the public, work and educational spheres. This is contradictory to results of other investigations showing that a high educational level would be a protective factor for violence [45,46]. This suggests that high educational level in women could be only a protective factor for sexual violence within the couple, whereas sexual violence experienced in other settings could be influenced by the society in which one lives. That is, living in a society with greater gender inequalities usually translates into a higher risk of sexual victimization experiences, compared to countries with a higher level of development in this area [91]. Communication and frequent contact with the family was positively associated with sexual violence at work and within a partnership. These results suggest that family networks, although they can be a space of trust and containment, often become a risk factor depending on the experiences lived within them, probably due to the dynamics and nature of family relationships and the influence this could have. For example, the presence of conflicts in the family of origin and the mistreatment experienced within them are predisposing factors for violence outside the family [91]. In Chile, the prevalence of domestic violence ranges from 30% to 80% [92], therefore, it is not surprising that some of the women we interviewed have had these experiences within their family of origin.

Participation in religious communities was positively associated with sexual violence in the partner and in education. This result could be explained due to the entire system of meanings that religious beliefs bring with them, guiding people’s actions on issues from marriage to sexuality, among other aspects. This set of beliefs constitutes the cognitive capital that gives meaning and action to these women facing experiences of sexual violence. Therefore, it is likely that this accumulation of religious beliefs prevents women from breaking the bond with an abusive partner in favor of maintaining the family as dictated by certain religious beliefs. In this sense, participation in religious communities could become a negative structural social capital due to a cognitive social capital based on rigid beliefs favoring inequality between genders.

High socioeconomic level, family networks and cognitive social capital associated with the acceptance of norms, values and attitudes towards traditional gender roles were positively associated with sexual violence only in the public sphere. Results suggest the role cognitive social capital could play specifically on the acceptance of cultural guidelines, legitimizing the domination of men over women, and how these beliefs permeate the different levels of interpersonal interaction in a society. Previous research has shown how the acceptance of traditional gender attitudes is related to a greater presence of violence in different settings towards women [93,94]. Likewise, family networks could become a negative structural capital when beliefs that validate gender inequalities are accepted at the family level.

In addition, we must not forget that sexual violence occurring in the public sphere brings with it a series of moral prejudices that often blame the victim of these episodes for not having taken the necessary precaution or care to prevent such experiences. Previous studies associated with rape myths reveal how the acceptance of these myths influences the perception of the victim’s guilt in episodes of sexual violence [15,93,94]. Finally, we did not expect to find a positive association with the high socioeconomic level and sexual violence in the public sphere, since previous research reported contrary results, although we must point out that these results were found only for sexual violence within the couple and it is likely that, when this violence occurs in the public sphere, other factors interfere that should be evaluated in future research.

### 4.3. Limitations

Regarding the limitations of the research, we can point out that although the public database was based upon information collected from women throughout Chile, it is likely that sexual violence is underrepresented due to beliefs and culture about this type of violence and, therefore, women do not report what really happens, especially in a stigmatized topic such as sexual violence against women in all areas. In addition, we cannot be sure that women’s understanding of sexual violence questions is the same as the researchers’ understanding.

Likewise, it would have been desirable to have evaluated other aspects associated with social, human and economic capital. However, by relying on a public database, we only had elements asked in that survey.

Finally, we suggest that future research delve deeper into the role of the different types of capital in sexual violence, not necessarily focusing on the victims. It would be desirable to also evaluate how these capitals influence the abusers who perpetrate the violent act.

## 5. Conclusions

Finally, the results allow us to advance our understanding of the role played by social, economic and human capital as protective or risk factors in sexual victimization of women in different interpersonal contexts. In this regard, the structural social capital of neighborhood networks is the main protective factor for sexual violence in all areas. These results allow us to question the role of family and friend networks, which, although they act as a protective effect in some areas, should not be taken for granted, since it is likely that the cognitive social capital associated with stereotyped gender beliefs may interfere with the protective effect of sexual violence. This may be due to the norms and values associated with the situation of sexual violence conceived by the members of these networks.

The structural social capital of networks with non-significant others was the main risk factor in sexual victimization in all areas. In this sense, blaming victims in situations of sexual violence is common in a society that accepts stereotyped gender roles, and because others may not understand the dynamics of this type of violence, experiences of this type are normalized.

Participation in networks of friends and family (structural social capital) also implies sharing certain social, cultural and family ideals that could imply maintaining and/or justifying experiences of sexual violence (when they happen), holding the victims responsible. Moreover, it is likely that the members of these networks do not want to become involved in the private lives of women, keeping themselves away from these situations.

Finally, although the different capitals analyzed have a dual action (protective/risk) when evaluated in different spaces (public, work, educational and couple), it does not mean that for the same relationship space they act as both a protective and risk factor, but rather that the same capital (social, economic and human) acts as a risk factor in one space, and in another, it can act as a protective factor. Thus, the educational level of the partner in the public sphere acts as a protective factor for sexual violence, but in another sphere, such as that of the partner, it acts as a risk factor. Therefore, although different capitals can act as both protective and risk factors for sexual violence, the individual and collective contribution to the prevention of sexual violence would be influenced by the context in which the experience of sexual violence occurs.

## Figures and Tables

**Table 1:** Sociodemographic variables of the sample.

Variable	N	%
Age		
15 to 19 years	384	23.1
20 to 25 years	656	39.4
26 to 30 years	625	37.5
Marital status		
Single	1496	89.8
Married	138	8.3
Civil union	15	0.9
Separated	9	0.5
Divorced	5	0.3
Widowed	2	0.1
Educational level		
No studies	2	0.1
Grade school	84	5.0
Secondary school	792	47.6
Technical professional	273	16.4
University education	506	30.4
Post-graduate studies	6	0.4
Socio-economic level		
High	128	7.7
Medium	688	41.3
Low	849	51.0

**Table 2:** Descriptive statistics of structural social capital.

Variable	Never	Once Per Week	Once Per Month	Once Per Year
	% (*n*)	% (*n*)	% (*n*)	% (*n*)
How often do you see or communicate with…?				
Immediate family	1.9 (32)	90.2 (1502)	5.3 (88)	2.4 (40)
People I consider family	7.7 (129)	77.7 (1293)	12.1 (202)	2.1 (35)
Friends	6.6 (110)	73.8 (1228)	16.5 (275)	2.8 (47)
Neighbors	24.1 (401)	55.3 (921)	15.6 (259)	4.9 (81)
Religious communities	69 (1149)	14.4 (239)	6.6 (110)	9.6 (160)
Others	75.7 (1260)	9.3 (155)	3.5 (58)	1.2 (20)

**Table 3:** Descriptive statistics of cognitive social capital.

Variable	Strongly Disagree	Disagree	Indifferent	In Agreement	Totally Agree
	% (*n*)	% (*n*)	% (*n*)	% (*n*)	
It is more appropriate for the man to be recognized as the head of the household.	29.2 (485)	48.8 (809)	6.8 (113)	12.2 (203)	2.9 (49)
Men should be responsible for family and household expenses.	25 (414)	43 (713)	6.4 (106)	19.7 (326)	6 (100)
Women should be responsible for the care of children instead of men.	30.1 (500)	51.9 (862)	5.4 (89)	9.9 (165)	2.7 (45)
Doing household chores (cleaning, washing, ironing, cooking) is a task more suitable for women than for men.	36.4 (604)	51.3 (852)	4.5 (75)	5.9 (98)	1.9 (31)
A wife/partner should not contradict her husband’s/partner’s opinion.	39.9 (662)	49.4 (819)	3.4 (57)	5.8 (97)	1.4 (24)
A woman may participate in a social activity, even if she does not have her husband’s/partner’s approval.	5.5 (91)	11.7 (193)	2.2 (37)	43.3 (716)	37.3 (618)
A woman can choose her friends, even if her husband does not like it.	3.9 (65)	7.6 (125)	1.8 (30)	44.7 (740)	42 (694)
A woman’s dress and make-up must be approved by her husband/partner.	38.8 (645)	52.5 (873)	2.6 (43)	4.5 (74)	1.6 (27)
A woman should have sexual relations with her husband/partner, even if she does not want to.	52.4 (869)	45.5 (755)	1 (16)	1 (16)	0.2 (3)
A woman should avoid dressing provocatively in order to avoid harassment.	37.1 (615)	48.2 (799)	3.5 (58)	8.5 (141)	2.8 (46)
Women should accept abuse for the sake of the family and their children.	53.6 (891)	44.6 (740)	0.8 (13)	0.9 (15)	0.1 (2)
If there are blows or mistreatment in the house, it is a matter to be solved in the family.	35.3 (580)	40.2 (660)	3.4 (56)	17.5 (287)	3.7 (60)
It is acceptable for a man to assault his partner in case of infidelity.	55.4 (921)	43.3 (719)	0.8 (13)	0.4 (6)	0.2 (3)

**Table 4:** Average coefficients between factors (types of capital) and areas of sexual violence.

Variables	Sexual Violation in Public Spaces	in Work Spaces	in Partnerships	in Educational Spaces
Socio-economic level	0.448	−0.372	−0.483	−0.057
Educational level	0.485	0.441	−0.081	0.049
Family networks	0.426	−1.191	−0.384	−0.472
Family similar networks	−0.092	1.411	0.206	−0.517
Friend networks	0.713	0.078	−0.567	0.866
Neighborhood networks	−0.244	−0.289	−0.392	−0.518
Religious org. networks	−0.275	−0.101	0.058	0.176
Other networks	0.051	0.822	0.464	0.454
Partner educational level	−0.100	0.471	0.652	0.368
Economic dependence	0.058	0.013	0.514	−0.748
Sexual stereotypes	0.064	−0.238	−0.144	−0.597

**Table 5:** Centrality indicators of degree and proximity of the nodes in the protection network.

Factor	Degree	Closeness
Neighborhood networks	1.00	0.89
Socio-economic level	0.75	0.77
Family network	0.75	0.77
Sexual stereotypes	0.75	0.77
Similar family networks	0.50	0.69
Religious organization networks	0.50	0.65
Education networks	0.25	0.59
Friend network	0.25	0.59
Economic dependency	0.25	0.59
Partner educational level	0.25	0.53
Other networks	0.00	0.34
Partner sexual violence	0.55	0.59
Sexual violence in educational sphere	0.55	0.59
Sexual violence in work place	0.45	0.55
Sexual violence in public places	0.36	0.52

**Table 6:** Risk network indicators of degree centrality and proximity of the nodes.

Factor	Degree	Closeness
Other networks	1.00	0.89
Educational level	0.75	0.83
Friend networks	0.75	0.83
Economic dependency	0.75	0.83
Educational level of partner	0.75	0.69
Similar family networks	0.50	0.65
Religious organizations network	0.50	0.65
Socio-economic level	0.25	0.62
Family network	0.25	0.62
Sexual stereotypes	0.25	0.62
Neighborhood network	0.00	0.34
Sexual violence in public spaces	0.64	0.63
Sexual violence in work spaces	0.55	0.59
Sexual violence in partnerships	0.45	0.55
Sexual violence in educational spaces	0.45	0.55

## Data Availability

The public database used in this research can be found at http://cead.spd.gov.cl/.
